# Three-Dimensional Interconnected Porous Partially Unzipped MWCNT/Graphene Composite Aerogels as Electrodes for High-Performance Supercapacitors

**DOI:** 10.3390/nano12040620

**Published:** 2022-02-12

**Authors:** Jun Zhou, Yuying Zheng, Dongyang Chen

**Affiliations:** College of Materials Science and Engineering, Fuzhou University, Fuzhou 350116, China; jzhou@fzu.edu.cn

**Keywords:** supercapacitor, aerogel, graphene, carbon nanotubes, nanoribbons

## Abstract

The self-restacking of graphene nanosheets inevitably compromises the electrochemical performance of conventional graphene-based materials. Herein, to solve this problem, we prepared a new type of three-dimensional porous aerogel with partially unzipped multiwalled carbon nanotubes inserted into graphene nanosheets via a reduction-reaction-induced self-assembly process. In the resulting aerogels, the inner carbon nanotubes (CNTs) tightly attach to the unzipped outer graphene nanoribbons (GNRs), which bridge with the graphene nanosheets. These interconnections bring them excellent electrical contact; the CNTs act as spacers to prevent the restacking of adjacent graphene nanosheets, and the abundant interconnected pores in the aerogels provide large channels for charge transfer. Accordingly, the aerogels exhibit a specific capacitance of 348.4 Fg^−1^ at a scan rate of 5 mVs^−1^, with capacitance retention remaining at 89.7% at a current density of 2 Ag^−1^ after 5000 cycles. The results show that the aerogels are promising electrode materials for supercapacitor applications.

## 1. Introduction

Nanocarbon materials have been widely researched as electrode materials because of their favorable electrical conductivity, high durability, low cost, and environmental friendliness. Among them, graphene-based materials possess high surface area, good electron mobility, and excellent mechanical properties compared with many of their counterparts, allowing them to be used as flexible electrode materials [1,2,3]. Although oxidation–reduction is a general method to produce graphene, the performance of the products is still not satisfactory [4]. The strong π–π interactions and van der Waals forces between adjacent graphene nanosheets trigger self-restacking of the two-dimensional (2D) nanostructure, significantly decreasing the effective surface area, and leading to a decline in electrochemical performance [5,6]. Accordingly, the main emphasis of the research in this field is focused on the efficient control of their restacking.

Many attempts have been made to solve this problem. One of the methods is to add carbon materials [7,8], metal oxides [9], or polymers [10] to act as spacers between adjacent graphene nanosheets. These spacers can prevent restacking to some extent compared with raw graphene; however, most of them always change the inherent properties of graphene, and bring complexity to the interface [11]. Previous studies have shown that CNTs as spacers can increase the electric conductivity of graphene [12], and their hollow, tube-shaped structures can improve the mechanical properties of graphene [13]. Moreover, GNRs are another form of workable spacer with similar planar structures to graphene nanosheets, and almost identical aspect ratios to CNTs [14,15]. However, CNTs, GNRs, and graphene nanosheets aggregate easily due to their similar properties, which are unfavorable for the performance of the products. To overcome this weakness, an effective solution is to design a rational connection mode between them.

In general, GNRs can be produced by the longitudinal unzipping of CNTs. The chemical oxidation reaction proceeds along the tube axis during unzipping, obtaining thin, ribbon-like products with particular physical and chemical properties [16,17]. The degree of the unzipping of CNTs can be controlled by the amount of oxidant. Mild oxidation makes multiwalled carbon nanotubes (MWCNTs) unzip only partially, with the inner CNTs remaining tightly attached to the unzipped outer GNRs (denoted as GONRs before reduction); the resulting products are called CNT@GNRs (denoted as CNT@GONRs before reduction), which possess the advantages of both CNTs and GNRs and, therefore, have better electrical conductivity and mechanical properties than pristine MWCNTs and fully unzipped GNRs derived from severe oxidations [11,18]. Meanwhile, the outer GNRs of CNT@GNRs can bridge with the graphene nanosheets due to their similar planar structures. This connection mode between CNTs, GNRs, and graphene nanosheets may bring them excellent electrical contact, achieving fast electron transfer.

Another method to prevent restacking of graphene is to build three-dimensional (3D) porous structures such as hydrogels or aerogels via a reduction-reaction-induced self-assembly process of graphene oxide (GO). The resulting graphene aerogels (GAs) are monolithic blocks or continuous fibers [19,20,21,22], presenting high porosity, low density, large surface area, and excellent strength and toughness [23,24,25]. The large numbers of 3D interconnected pores in GAs prevent the restacking of graphene nanosheets and increase the contact area between electrolyte and electrode; they also provide more transport channels for ions, creating favorable conditions for electron transfer [26,27].

Unlike graphene/CNT aerogels or graphene/GNR aerogels, which are simple mixtures of graphene nanosheets and CNTs or GNRs and, thus, lack effective intrinsic connections, in this work, we inserted CNT@GNRs into graphene nanosheets uniformly in order to form 3D porous aerogels, which were named CNT@GNR@GAs. The outer ribbons from CNT@GNRs could highly integrate into graphene nanosheets; meanwhile, the remaining inner tubes served as both spacers against the restacking of graphene nanosheets and frameworks to prevent aerogels from shrinking or collapsing during the drying treatment. The aerogels were formed through a facile reduction-reaction-induced self-assembly process, which could well maintain the uniformity of the aerogels as well as remove the oxygen-containing functional groups of GO [28]. With such unique structural features, the CNT@GNR@GAs exhibited quick and sufficient electron and charge transfer, giving them excellent electrochemical performance.

## 2. Materials and Methods

### 2.1. Materials

Flake graphite powders (99.9%), MWCNTs (99.9%), and sodium ascorbate (99.9%) were purchased from Aladdin Chemical Co. Ltd, Shanghai, China. H_2_SO_4_ (98%), H_3_PO_4_ (85%), KMnO_4_ (99.5%), H_2_O_2_ (30%), HCl (36%), HNO_3_ (65%), ethanol (99%), and KOH (99%) were purchased from Sinopharm Chemical Reagent Co. Ltd, Shanghai, China. Nickel foams were purchased from YiYang Foammetal New Material Co. Ltd, Yiyang, China.

### 2.2. Synthesis of GO and CNT@GONRs

GO was synthesized via a modified Hummers method [29]. Flake graphite powders (3.0 g) were put into a solution of concentrated H_2_SO_4_ (135 mL) and H_3_PO_4_ (85%, 15 mL) and stirred for 30 min at 0 °C, and then KMnO_4_ (18 g) was added slowly and stirred for another 30 min. After that, the reaction mixture was heated to 50 °C and stirred for 12 h. The cooled mixture was then poured into deionized water (1 L) containing a moderate amount of H_2_O_2_ (30%) and stirred until the solution turned bright yellow. After filtering, the remaining solid was washed with HCl (5%), followed by repeated centrifugation with deionized water to remove the acid and impurities. The resulting GO was dispersed in deionized water by sonication for 2 h; the concentration of the GO dispersion was 4 mg mL^−1^.

CNT@GONRs were synthesized by partially unzipping the MWCNTs [18]. Pristine MWCNTs (0.15 g) were put into concentrated H_2_SO_4_ (36 mL) and stirred for 2 h at room temperature, followed by the addition of H_3_PO_4_ (85%, 4 mL), and stirred for another 15 min; then, KMnO_4_ (0.6 g) was slowly added into the mixture over the course of 1 h. The reaction mixture was heated to 65 °C and stirred for 2 h, and then cooled to room temperature. Thereafter, the mixture was poured into icy deionized water (100 mL) containing H_2_O_2_ (30%, 5 mL) and left to stand for 14 h. The supernatant was filtered and the precipitate was washed with HCl (5%) and a large amount of deionized water via vacuum suction filtration. The final CNT@GONR dispersion was concentrated to 4 mg mL^−1^.

For comparison, GONRs (fully unzipped MWCNTs) were prepared via a similar process to CNT@GONRs, except that the amount of KMnO_4_ was 1.2 g [30]. Another control sample used intact CNTs; in order for them to disperse uniformly, they should be treated with acid first to increase their hydrophilicity. Pristine MWCNTs (0.15 g) were put into HNO_3_ (65%, 12 mL) and stirred for 12 h at 120 °C; the products were washed with deionized water and concentrated to 4 mg·mL^−1^.

### 2.3. Synthesis of CNT@GNR@GAs

**Scheme 1:**
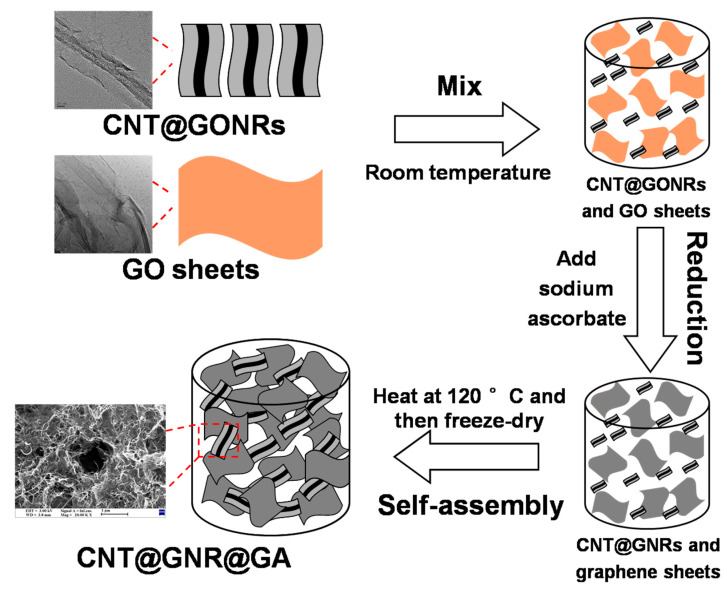
Illustration of the synthetic process for CNT@GNR@GAs.

The CNT@GNR@GAs were synthesized through a reduction-reaction-induced self-assembly process, as shown in Scheme 1. The above-mentioned GO dispersion (10 mL) and CNT@GONR dispersion (10 mL) were mixed and stirred gently for 30 min at room temperature. Sodium ascorbate aqueous solution (1 mol·L^−1^, 1 mL) was added to the mixture and heated to 120 °C in an oil bath for 2 h without stirring. The resulting hydrogels were washed with deionized water and dipped into ethanol solution (20%) for 6 h, and then freeze-dried for 24 h.

For comparison, GAs were prepared via the same process without adding CNT@GONRs. Graphene/GNR aerogels (GNR/GAs) and graphene/CNT aerogels (CNT/GAs) were also prepared; the processes were similar to that used to prepare CNT@GNR@GAs, except that the CNT@GONRs were changed to GONRs and acidified MWCNTs, respectively.

### 2.4. Characterizations

Transmission electron microscopy was conducted on a Tecnai F30 microscope (FEI, Hillsboro, OR, USA). X-ray photoelectron spectroscopy was measured by an ESCALAB 250 spectrometer (Thermo Fisher Scientific, Waltham, MA, USA). Fourier-transform infrared spectra were recorded by a Nicolet 5700 FT-IR spectrometer (Thermo Fisher Scientific, Waltham, MA, USA). X-ray diffraction was measured on an Ultima III (Rigaku, Tokyo, Japan) with Cu Kα radiation. Scanning electron microscopy was performed using a Supra55 microscope (Carl Zeiss, Oberkochen, Germany). Raman spectra were recorded using an inVia reflex Raman spectrometer (Renishaw, London, UK) with laser excitation at 514 nm. Nitrogen adsorption/desorption measurements were conducted on an ASAP 2460 surface area and porosity analyzer (Micromeritics, Atlanta, GA, USA).

### 2.5. Electrochemical Measurements

A thin slice (1 cm × 1 cm × 0.2 cm) was separated from the as-prepared aerogels and dipped into a 6 M KOH aqueous solution at room temperature for one night. The slice was sandwiched by nickel foams and compressed under 25 MPa for 5 min. Subsequently, the aerogels on nickel foams were then connected to a platinum wire, while another platinum wire and Ag/AgCl were used as the counter and reference electrodes, respectively. All electrochemical measurements were performed in a three-electrode system using a CHI660D electrochemical workstation (Chenhua, Shanghai, China); the electrolyte was a 6 M KOH aqueous solution. The electrochemical performances were characterized by cyclic voltammetry (CV), galvanostatic charge–discharge test (GCD), and electrochemical impedance spectroscopy (EIS). CV and GCD were conducted in the potential range of 0–0.5 V, while EIS was conducted in the frequency range from 100 kHz to 1 Hz at an amplitude of 5 mV, referring to the open-circuit potential.

The specific capacitance was calculated from the CV curves according to the following equation: *C*_CV_ = $\left(\int^{\;}IdV\right)/\left(m\kappa \Delta V\right)$, where *I* is the response current (A), *m* is the mass of aerogels (g), *κ* is the scan rate (mVs^−^^1^), and Δ*V* is the potential range (V). The specific capacitance could also be obtained from the GCD curves according to the equation *C*_GCD_ =2(IΔt)/(mΔV), where *I* is the constant discharge current (A), Δ*t* is the discharge time (s), *m* is the mass of the aerogels (g), and Δ*V* is the discharge potential window (V) excluding the *IR* drop.

## 3. Results and Discussion

**Figure 1:**
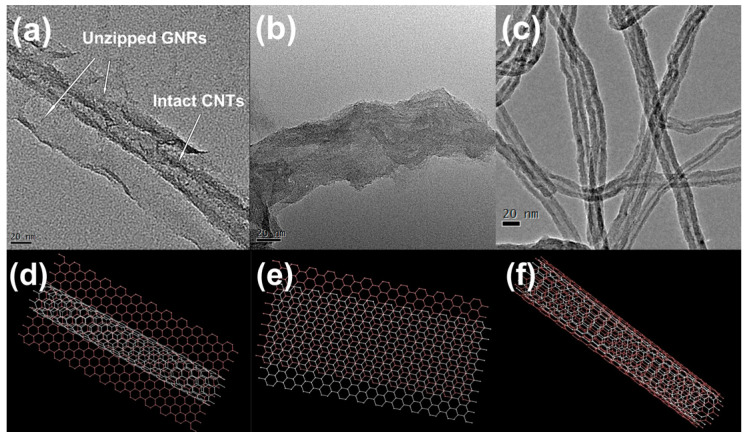
(**a**–**c**) TEM images and (**d**–**f**) structure diagrams of the CNT@GONRs, GONRs, and MWCNTs.

The microstructure and morphology of the CNT@GONRs, GONRs and pristine MWCNTs were characterized by transmission electron microscopy (TEM). As shown in Figure 1, the different amounts of KMnO_4_ caused significant differences in the resultant unzipped MWCNTs. Studies have shown that the theoretical limit of consumption of KMnO_4_ for complete oxidation of all alkenes is 4.4 times the weight of the MWCNTs (440 wt%) [18]. When the amount of KMnO_4_ was400 wt%, the MWCNTs were partially unzipped (CNT@GONRs), as shown in Figure 1a.The outer walls of the MWCNTs were unzipped to ribbons, stacking together with the intact inner CNTs. The ideal structure is shown in Figure 1d. After increasing KMnO_4_ to 800 wt%, the MWCNTs were fully unzipped (GONRs), with the completely exfoliated GNRs stuck together, as shown in Figure 1b,e. For comparison, the pristine MWCNTs are shown in Figure 1c,f.

**Figure 2:**
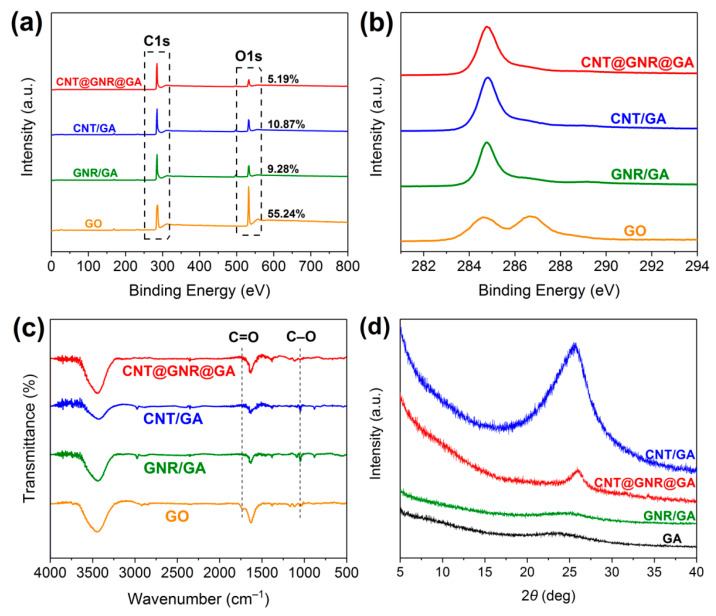
(**a**) XPS large survey spectra, (**b**) XPS C1s spectra, (**c**) FTIR spectra, and (**d**) XRD patterns of the resulting aerogels.

X-ray photoelectron spectroscopy (XPS) was used to analyze the element species and chemical attributes of the different samples. In the XPS C1s spectra of the CNT@GONRs (Figure S1a), the three peaks at 286.2, 287.1, and 288.2 eV—corresponding to C–O (epoxy/hydroxyl), C=O (carbonyl), and O–C=O (carboxyl) [31], respectively—reveal that an abundance of oxygen-containing functional groups in the form of *sp*^3^-hybridized saturated carbons was generated during the unzipping of the MWCNTs. The spectra were similar to GO, as reported in [7]. After reduction, as shown in the spectrum of the CNT@GNR@GA, the peak intensity of C=C (284.5 eV) rose dramatically, while that of C–O, C=O, and O–C=O decreased sharply, indicating the removal of oxygen-containing functional groups. The peak of π–π* (289.3 eV, Figure S1b) appeared, revealing the recovery of aromaticity and saturation. The *sp*^2^ carbon network was re-established during the reduction process [32]. In the large survey spectra (Figure 2a), only carbon and oxygen could be observed. The oxygen contents of the CNT@GNR@GA, CNT/GA, and GNR/GA were 5.19%, 10.87%, and 9.28%, respectively. These oxygen content decreased significantly compared with the GO (55.24%), meaning that most of the oxygen elements were removed during the reduction process, which could also be confirmed by the high-resolution C1s spectra of these aerogels (Figure 2b). When using various unzipped MWCNTs as precursors, the oxygen content within the corresponding aerogels was slightly different. This can be explained by the distinct degree of oxidation and the preservation of some intact internal CNTs.

The evolution of functional groups during the reduction process was also confirmed by Fourier-transform infrared (FTIR) spectroscopy, as shown in Figure 2c. In the spectrum of the GO, the two characteristic peaks at approximately 1735 and 1049 cm^−1^ correspond to C=O and C–O stretching peaks, respectively; these groups are the result of oxidation [33,34]. After reduction, the oxidation peaks were decreased in the spectra of the GNR/GA, CNT/GA, and CNT@GNR@GA, leaving only the C=C (approximately 1624 cm^−1^), reflecting the oxygen-containing functional groups were removed.

X-ray diffraction (XRD) patterns provided further evidence for the reduction. The structures of various degrees of unzipped MWCNTs are shown in Figure S2. The diffraction peaks appeared at around 2*θ* = 25.2°, corresponding to the pristine MWCNTs (002) plane, similar to the peaks of the reduced GO (rGO). When the pristine MWCNTs were partially unzipped, the resultant CNT@GONRs showed a new diffraction peak at around 11°; meanwhile, the acidified MWCNTs also showed a weak peak at the same 2*θ* value because of the mild oxidation, revealing that the relative intensity of the peak at 11° was increased with the level of oxidation. The XRD patterns of the corresponding aerogels are shown in Figure 2d. All of them showed a predominant broad peak at around 25.2°, while the typical diffraction peak of the GO or GONR at around 11° did not occur, proving that a reduction process occurred, and the result was consistent with those of XPS and FTIR [35,36]. These weak and broad diffraction peaks reflect that the aerogels maintained the 3D porous framework, but also the highly dense structure, corresponding to long structure disordering but short structure ordering [37]. Compared with the GA and GNR/GA, which only contained graphene nanosheets, the CNT/GA and CNT@GNR@GA contained intact CNTs, which may be the reason why the diffraction angle and the intensity of the peaks showed a slight difference. The entire CNT@GNR@GA presented a lower degree of restacking of graphene and a larger extent of structural porosity, because the retained intact CNTs served as spacers between the graphene layers and enlarged the interspaces [38]. The average number of graphene layers can also be calculated by fitting the XRD data with the structural analysis software TOPAS (Bruker AXS) [32]; however, in our composite system, the fitting of the XRD peaks may be difficult.

**Figure 3:**
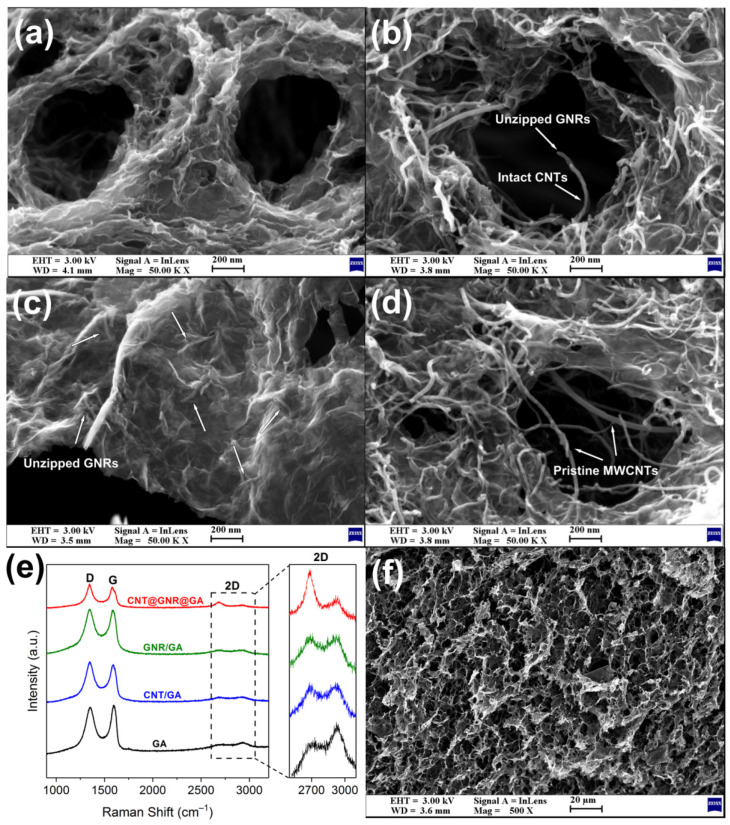
SEM images of (**a**) GA, (**b**) CNT@GNR@GA, (**c**) GNR/GA, and (**d**) CNT/GA. (**e**) Raman spectra of the resulting aerogels; (**f**) cross-sectional SEM image of CNT@GNR@GA.

The morphology and microstructure of aerogels were characterized by scanning electron microscopy (SEM). Figure 3a–d correspond to GA, CNT@GNR@GA, GNR/GA, and CNT/GA, respectively. The graphene nanosheets interwove together and formed pores, where the introduced CNTs or GNRs embedded or adhered uniformly within the graphene walls. Most of the CNTs and GNRs were distributed individually, without forming aggregations (except for a small number of aggregations in GNR/GA), indicating that they were mixed evenly with the GO sheets in the reaction process. The introduced CNTs and GNRs served as spacers to prevent the graphene nanosheets from restacking [37]. In addition, they could also act as bridges to connect the graphene nanosheets around them. The strong adhesion between the CNTs/GNRs and graphene nanosheets came from their similar morphology and their π–π interactions [30]. Unlike the flat morphology and 2D structure of GNRs, the CNTs could act as frames to sustain graphene aerogels, and their flexibility mitigated the shrinkage of the aerogels under the capillary compression during freeze-drying [7]. It is worth noting that the CNT@GNR@GA contained both GNRs and CNTs; on the one hand, the wing-like GNRs adhered at the graphene nanosheets tightly; on the other hand, the support of the intact CNTs prevented the aerogels from collapsing, and the monolithic aerogels showed a dense and flexible structure. Compared with GNR/GA (containing only GNRs), CNT/GA (containing only CNTs), and GA, the graphene nanosheets in the CNT@GNR@GA were thinner and more transparent, as shown in the SEM images.

These results were also confirmed by Raman spectra (514 nm laser excitation), as shown in Figure 3e. The 2D band at ~2700 cm^−1^ of CNT@GNR@GA was single and sharp, showing typical single-layer graphene; the 2D bands of the GNR/GA and CNT/GA were much broader, proving that the samples comprised 2–5 layers; the spectrum of the GA was similar to that of bulk graphite, indicating that there were more than 5; moreover, the gradual increase in the number of layers led to an upshift of 2D peaks [39]. In all of the samples, two predominant peaks at around 1584 and 1350 cm^−1^ corresponded to the G and D bands, respectively. The similar G and D bands confirmed that the structure of the CNTs was not destroyed during the unzipping process. The GA also contained similar bands, consistent with previous reports with respect to chemically rGO [31,40]. The I_D_/I_G_ intensity ratio could describe the degree of defect in carbon materials in a straightforward manner. The GNR/GA, CNT/GA, and CNT@GNR@GA exhibited higher I_D_/I_G_ than the GA because of the introduction of CNTs and GNRs, suggesting the increasing level of the disorder [41].

Figure 3f shows the cross-section SEM image of the monolithic CNT@GNR@GA. The structure was porous and dense, and the highly wrinkled graphene nanosheets and intact CNTs were interwoven together, with pore widths in the range from hundreds of nanometers to tens of micrometers. Additionally, the cross-section SEM images of the CNT/GA, GNR/GA, and GA are shown in Figure S3a–c, respectively, with similar structures.

**Figure 4:**
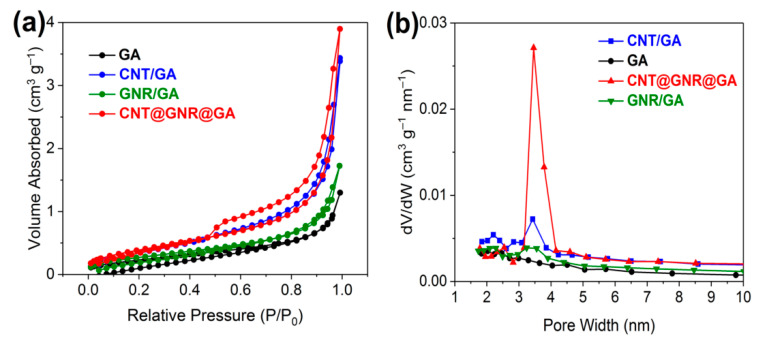
(**a**) Nitrogen sorption isotherms; (**b**) pore size distribution curves of the resulting aerogels.

In addition to SEM, the nitrogen adsorption/desorption isotherms were used to analyze the porous microstructures and Brunauer–Emmett–Teller (BET) specific surface areas of the resulting aerogels, as shown in Figure 4a. According to the International Union of Pure and Applied Chemistry (IUPAC) classification, the GA and GNR/GA exhibited Type II isotherms, indicating that the hierarchical pore structures were mainly associated with macropores, and nearly none of them were associated with micropores or mesopores. The CNT/GA exhibited some characteristics of Type II and IV isotherms; a less obvious H3 hysteresis loop appeared at the relative pressure of 0.7–1.0, indicating that very limited numbers of micropores and mesopores existed in the texture of the aerogel. The CNT@GNR@GA exhibited Type IV isotherms with an obvious H3 hysteresis loop, suggesting an open, wedge-shaped structure with a certain amount of mesopores. These mesopores might be constructed by the compact stacking of the wrinkled graphene nanosheets and flexible CNT@GNRs. The sharp rise at the relative pressure of 0.9–1.0 signified no saturation adsorption due to the uneven distribution of pore size [7]. The specific surface areas of the CNT@GNR@GA and GNT/GA were calculated to be 36.4 m^2^g^−^^1^ and 33.3 m^2^g^−^^1^, respectively—much higher than those of the CNR/GA (23.0 m^2^g^−^^1^) and GA (20.1 m^2^g^−^^1^), indicating that the introduced CNTs prevented the restacking of graphene nanosheets and created more interspace. Likewise, CNT@GNR@GA and GNT/GA also had a significantly higher pore volume than GNR/GA and GA, as shown in Table S1. The corresponding pore size distributions were further plotted, as shown in Figure 4b. The results were consistent with the above-mentioned analyses of the nitrogen adsorption/desorption isotherms. It is noteworthy that the CNT@GNR@GA exhibited small mesopores with diameters in the range of 3–4.5 nm. The mesoporous structure could promote ion transport and adsorption at the interface between the electrode and electrolyte [42].

**Figure 5:**
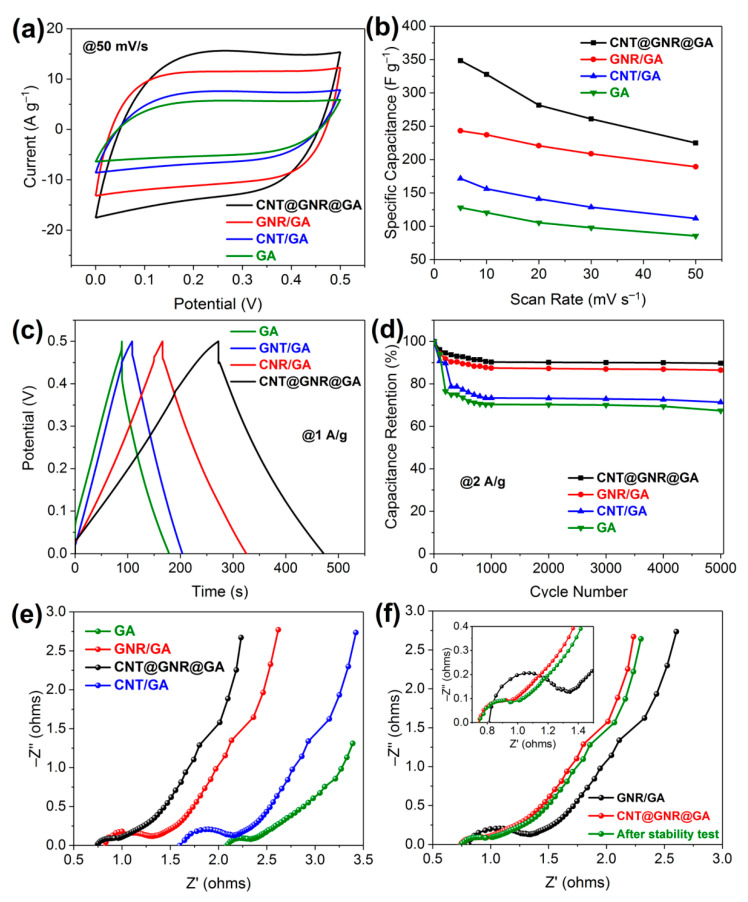
Electrochemical performance of aerogels in 6 M KOH: (**a**) CV curves at a scan rate of 50 mVs^−1^; (**b**) calculated specific capacitance at various scan rates; (**c**) the GCD curves at a current density of 1 Ag^−1^; (**d**) cyclic stability obtained from the GCD test at a current density of 2 Ag^−1^; (**e**) Nyquist plots collected by EIS; (**f**) Nyquist plots for CNT@GNR@GA before and after cyclic stability test and GNR/GA.

To investigate the electrochemical performances, the electrodes prepared from the resulting aerogels were tested in an aqueous electrolyte of 6 M KOH at room temperature. Figure S4a–d show the CV curves of the corresponding electrodes (CNT@GNR@GA, GNR/GA, CNT/GA, and GA, respectively) at various scan rates, from 5 to 50 mVs^−1^. The CV curves of all of the samples exhibited a nearly rectangular shape without redox peaks—especially at 5 mVs^−1^—revealing that the electrode interface possessed excellent charge transportation according to the mechanism of the double-layer capacitors. When the scan rate increased, the current response increased accordingly, along with various degrees of distortion of the shapes. At the same scan rate (50 mVs^−1^), in Figure 5a, we can see that the CV curve of the CNT@GNR@GA had less distortion than those of the other three aerogels, and the capacitive behavior of the CNT@GNR@GA was remarkably better than that of the other aerogels. As shown in Figure 5b, the specific capacitance of the CNT@GNR@GA was 348.4 Fg^−1^ at 5 mVs^−1^. In comparison, the specific capacitance of the GNR/GA, CNT/GA, and GA at the same scan rate was to 243.2 Fg^−1^, 171.8 Fg^−1^, and 128.1 Fg^−1^, respectively—similar to previous reports [30]. Figure S4e shows the deviation and average specific capacitance regardless of the scan rate. The introduced CNT@GNRs brought a pronounced increase in the specific capacitance, because the interconnections between the graphene nanosheets, GNRs, and CNTs in the CNT@GNR@GA offered more efficient electron transport channels. With the scan rate increased to 50 mVs^−1^, the specific capacitance of the CNT@GNR@GA decreased to 224.9 Fg^−1^, which was ~64.6% of the initial value.

The GCD curves of the above-mentioned electrodes at a current density of 1 Ag^−1^ are shown in Figure 5c. The GCD curves of the composite aerogels (CNT@GNR@GA, GNR/GA, and CNT/GA) appeared to be a nearly symmetric triangular shape, which is also a remarkable characteristic of double-layer capacitors, indicating that these electrodes had excellent charge–discharge performance and electrochemical reversibility. Comparatively, the GCD curve of the pure GA showed more apparent distortion of the triangle and a larger *IR* drop than the composite aerogels. The results suggest that the introduced GNRs or CNTs acted not only as spacers against the self-stacking of graphene nanosheets, but also as bridges to connect adjacent graphene nanosheets, achieving a small internal resistance with high faradaic efficiency [43,44]. The specific capacitance of CNT@GNR@GA calculated from the GCD curve was 313.2 Fg^−1^ at the current density of 1 Ag^−1^, which was higher than that of the GNR/GA (259.7 Fg^−1^), CNT/GA (165.9 Fg^−1^), and GA (124.7 Fg^−1^). The calculations were consistent with those of CV curves. The cyclic stability of the electrodes was also tested at the current density of 2 Ag^−1^, as shown in Figure 5d. The result showed that the capacitance retention of CNT@GNR@GA was 89.7% after 5000 charge–discharge cycles, and the specific capacitance tended to be stable after 700 cycles, demonstrating good electrochemical stability. Furthermore, the cyclic stability of the CNT@GNR@GA was also better than that of the other three aerogels.

The EIS was applied as a supplementary technique to study the electrochemical performances of aerogels. The typical Nyquist plots are shown in Figure 5e. The semicircle curve in the high-frequency region reflects the ion transport at the electrode/electrolyte interface, referred to as the charge-transfer resistance; the slope of the 45° portion of the curve reflects the electrolyte diffusion and transport within the electrode, referred to as the Warburg impedance, while the vertical curve in the low-frequency region reflects the ideal capacitive behavior. Furthermore, the intercept of the curve at the Z’ axis represents the equivalent series resistance (ESR), including the internal resistance of the material, contact resistance, electrolyte resistance, etc. [45,46]. As shown in Figure 5e, the ESR of the CNT@GNR@GA, GNR/GA, CNT/GA, and GA was 0.74 Ω, 0.82 Ω, 1.59 Ω, and 2.08 Ω, respectively. With the introduction of CNT@GNRs, the ESR of the CNT@GNR@GA was lower than that of the other three aerogels. Meanwhile, the Warburg impedance decreased, revealing that the CNT@GNR@GA possessed better rate capabilities in contrast with the other three aerogels. In the low-frequency region, the slope of the curve of the CNT@GNR@GA was higher than that of the other three aerogels, proving that the capacitive behavior of the CNT@GNR@GA was better. In the high-frequency region, the CNT@GNR@GA presented an obviously smaller semicircle than the other three aerogels, illustrating a lower charge-transfer resistance. In summary, the CNT@GNRs changed the microstructures of the aerogels and increased their diffusion and transport capabilities for electrons and ions, thus improving their electrochemical performances significantly. This result coincided with the analyses of CV and GCD.

The Nyquist plots for the CNT@GNR@GA before and after the cyclic stability test are shown in Figure 5f. After 5000 cycles, the ESR of the CNT@GNR@GA increased marginally; the slope of the curve dropped mildly in the low-frequency region, while the charge-transfer resistance and the Warburg impedance remained virtually unchanged, revealing that the microstructure of the monolithic aerogels was stable, with the electrochemical performances being impaired slightly after the charge–discharge cycles. Furthermore, the electrochemical performance of CNT@GNR@GA after 5000 cycles was still better than that of GNR/GA. These results are consistent with the analyses of Figure 5d.

**Figure 6:**
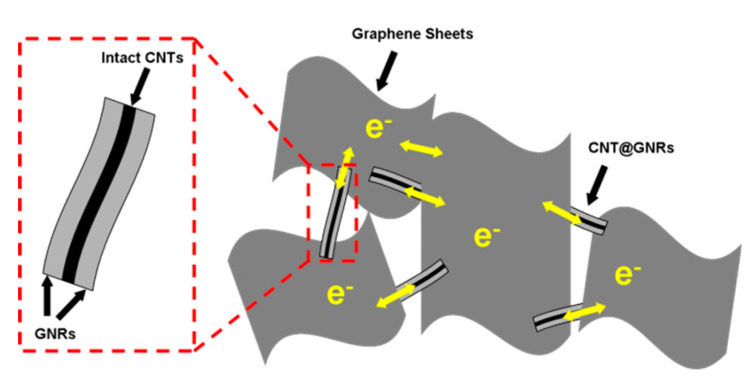
The interconnected structure of CNT@GNR@GA to enhance electron transfer.

The excellent electrochemical performance can be attributed to the unique microstructural features of the CNT@GNR@GA, as illustrated in Figure 6. In the CNT@GNR@GA, the inner CNTs of the CNT@GNRs acted as spacers to prevent the restacking of the adjacent graphene nanosheets; meanwhile, and the graphene nanosheets served as spacers to impede the aggregation of the adjacent CNT@GNRs. In addition, the unzipped outer GNRs acted as bridges to connect the CNT@GNRs and the graphene nanosheets, which could ensure a quick and adequate electron transfer among the monolithic aerogels. By contrast, the graphene nanosheets restacked seriously in the GA due to the absence of spacers (Figure 3a); there were adequate GNRs acting as spacers in the GNR/GA, but the GNRs did not prevent the restacking of the graphene nanosheets effectively because of their 2D structure and similar morphology to the graphene nanosheets (Figure 3c). In the CNT/GA, the CNTs and the graphene nanosheets assembled uncontrollably, accompanying strong π–π interactions between them; hence, they aggregated and formed boundaries easily [47], and these boundaries inhibited the transfer of electrons (Figure 3d).

Moreover, the CNT@GNR@GA possessed both rigid CNTs and flexible GNRs/graphene nanosheets, achieving a combination of strength and toughness of the monolithic aerogels. The CNTs served as a framework to mitigate the shrinkage of the hydrogels and prevent the aerogels from collapsing during the freeze-drying treatment, thus obtaining porous aerogels. The abundant pore structure of the aerogels was favorable for electrolyte infiltration. Meanwhile, the GNRs/graphene nanosheets made the aerogels sturdy enough to maintain their porous structure when the pores were filled with electrolytes. The nanoscale pores had a homogeneous distribution throughout the CNT@GNR@GA (Figure 4b). Compared with the nonporous materials, these plentiful pores enabled fast electron and charge transport in multiple dimensions and multiple directions among the monolithic aerogels [48]. Therefore, the CNT@GNR@GA possessed better electrochemical performance than the other three aerogels, even though the four aerogels had similar compositions and structures. As a result, the CNT@GNR@GA could be an excellent electrode material for double-layer supercapacitors.

## 4. Conclusions

In summary, we fabricated a type of 3D porous aerogel by integrating partially unzipped MWCNTs into graphene nanosheets via a reduction-reaction-induced self-assembly method. The resulting CNT@GNRs, derived from partially unzipped MWCNTs, were composed of the intact CNTs and the unzipped GNRs, which stuck together tightly. The CNT@GNRs acted as bridges and spacers to connect the adjacent graphene nanosheets but prevent them from restacking. The reduction-reaction-induced self-assembly method could greatly sustain the uniformity and porosity of the aerogels during the reduction process. The resulting aerogels exhibited a mesoporous structure, with pore widths in the range of 3–4.5 nm. The interconnected structure combined with the porous structure; the monolithic aerogels possessed excellent electrical contact, abundant electron transfer channels and, thus, better electrochemical performance. As a result, the specific capacitance of the aerogels was 348.4 Fg^−1^ at a scan rate of 5 mVs^−1^, with an ESR of 0.74 Ω. After 5000 cycles, the capacitance retention remained at 89.7% at a current density of 2 Ag^−1^. The results show that the CNT@GNR@GA can be one kind of promising electrode material. The performance of the CNT@GNR@GA may be further improved by loading conductive polymers or metal oxides into the aerogels for additional pseudocapacitance.

## Data Availability

The data presented in this study are available on request from the corresponding author.

## Supplementary Materials

The following supporting information can be downloaded at https://www.mdpi.com/article/10.3390/nano12040620/s1. Figure S1: XPS C1s spectra of (a) CNT@GONRs and (b) CNT@GNR@GA; Figure S2: XRD patterns of rGO, pristine MWCNTs, acidified MWCNTs, and CNT@GONRs; Figure S3: Cross-sectional SEM images of (a) CNT/GA, (b) GNR/GA, and (c) GA; Figure S4: CV curves at various scan rates of (a) CNT@GNR@GA, (b) GNR@GA, (c) CNT/GA, and (d) GA, (e) deviation and average specific capacitances; Table S1: Comparison of the BET surface area and pore volume of the resulting aerogels.
