# Supplementary material for: Three-Dimensional Interconnected Porous Partially Unzipped MWCNT/Graphene Composite Aerogels as Electrodes for High-Performance Supercapacitors
Source: Nanomaterials (Basel). 2022 Feb 12;12(4):620. doi: 10.3390/nano12040620 (PMC8874648; doi:10.3390/nano12040620)
Supplement: Supplementary file 1 [file nanomaterials-12-00620-s001.zip › nanomaterials-1548766-supplementary.pdf]

nanomaterials

MDPI

Supplementary Materials

Three-Dimensional Interconnected Porous Partially Unzipped MWCNT/Graphene Composite Aerogels as Electrodes for High-Performance Supercapacitors

Jun Zhou, Yuying Zheng * and Dongyang Chen *

College of Materials Science and Engineering, Fuzhou University, Fuzhou 350116, China; jzhou@fzu.edu.cn
* Correspondence: yyzheng@fzu.edu.cn (Y.Z.); dongyang.chen@fzu.edu.cn (D.C.)

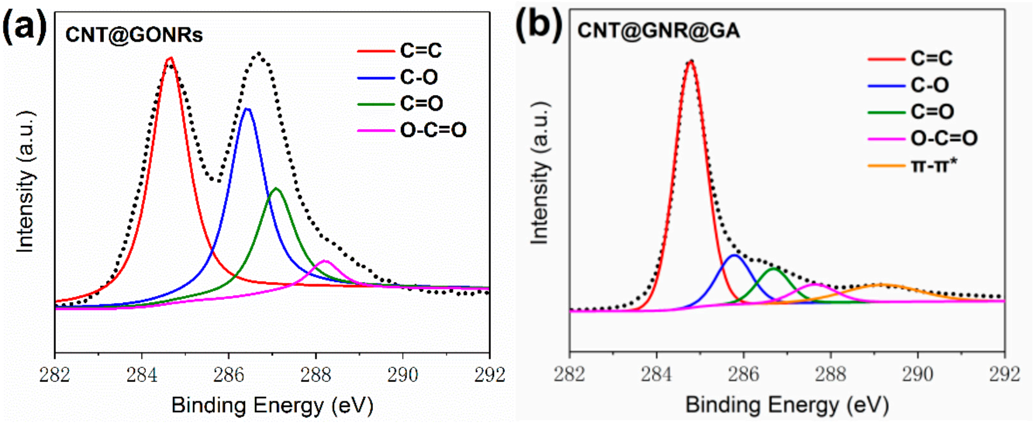

Figure S1. XPS C1s spectra of (a) CNT@GONRs, **(b)** CNT@GNR@GA.

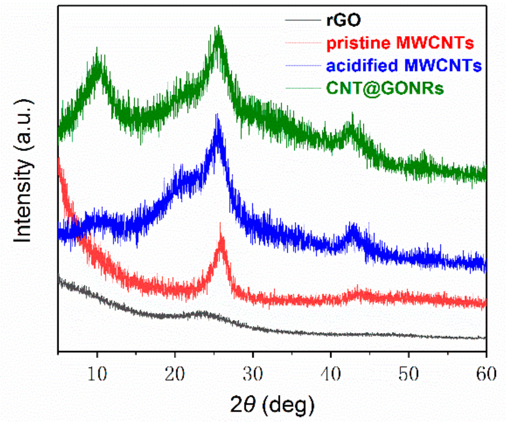

Figure S2. XRD patterns of rGO, pristine MWCNTs, acidified MWCNTs, and CNT@GONRs.

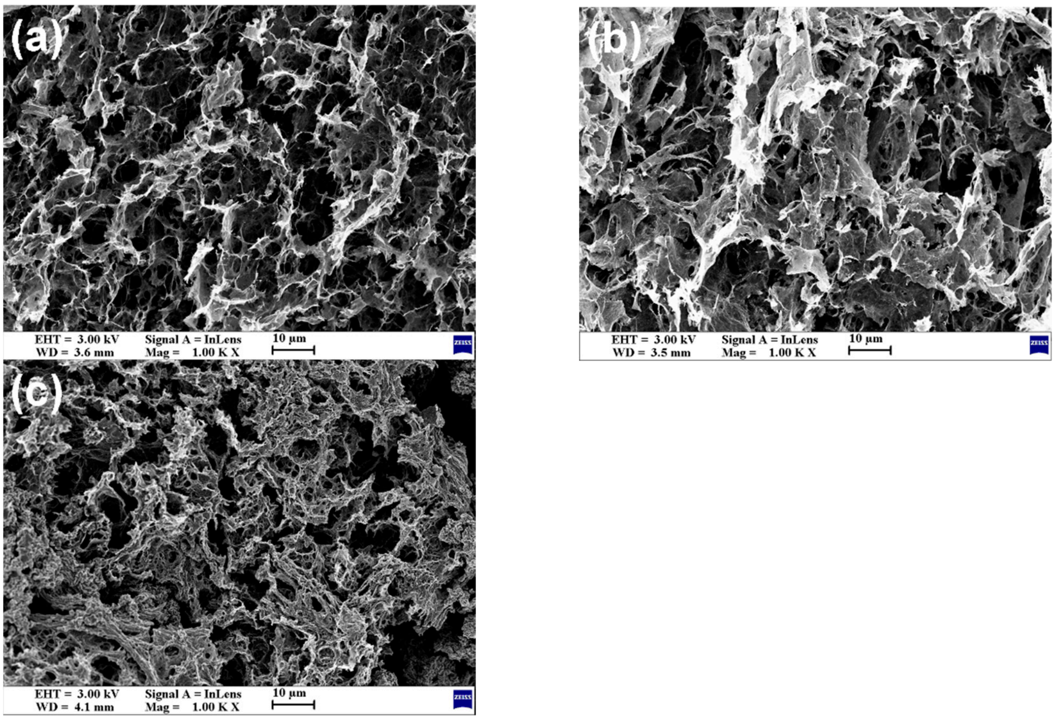

Figure S3. Cross-sectional SEM images of**(a)** CNT/GA,**(b)**GNR/GA, and**(c)** GA.

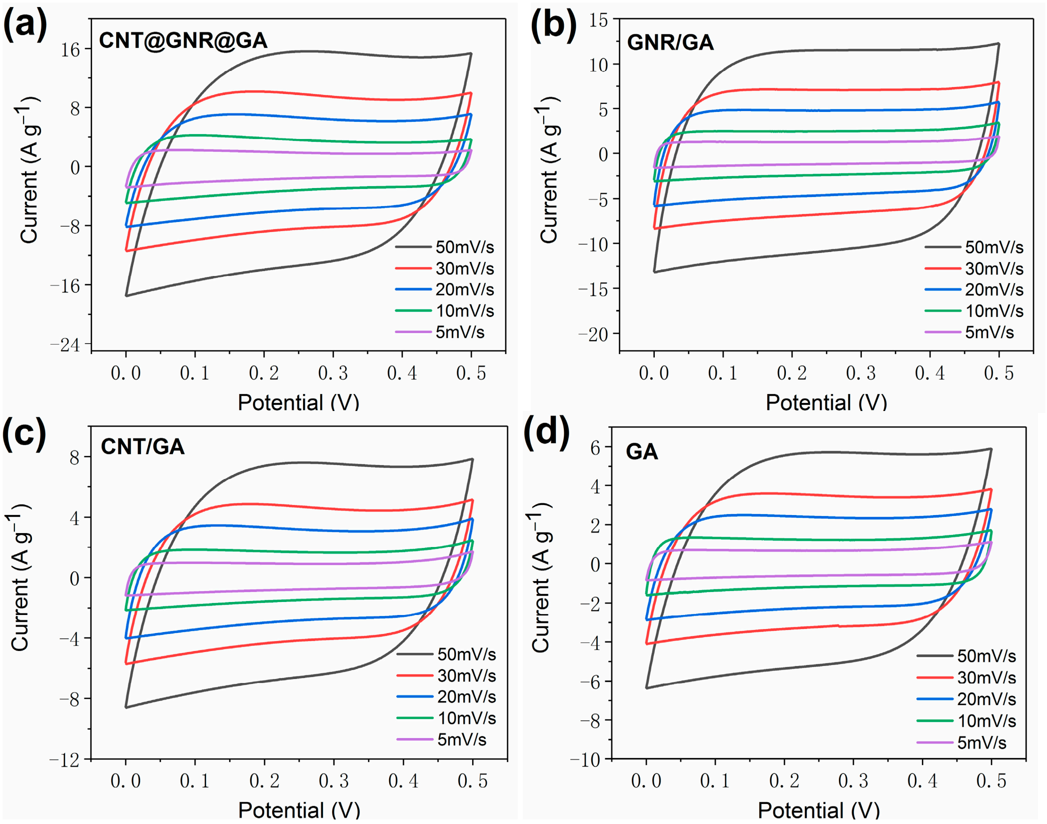

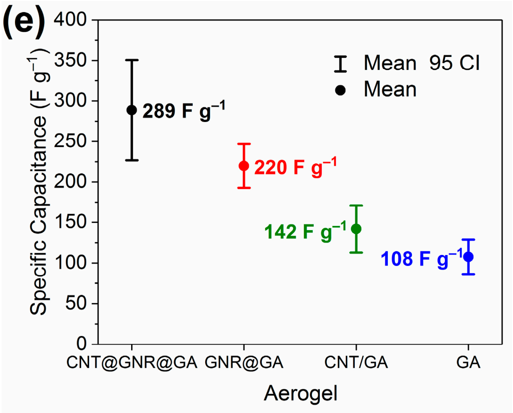

Figure S4. CV curves at various scan rates of **(a)** CNT@GNR@GA, **(b)** GNR@GA,**(c)** CNT/GA, **(d)** GA, **(e)**deviation and average specific capacitances.

Table S1. Comparison of the BET surface area and pore volume of the resulting aerogels.

Sample	BET surface area (m^2/g)	BJH Adsorption cumulative volume of pores (cm^3/g)	BJH Desorption cumulative volume of pores (cm^3/g)
GA	20.0905	0.046035	0.042245
GNR/GA	22.9722	0.060928	0.058350
CNT/GA	33.2858	0.120791	0.117129
CNT@GNR@GA	36.4287	0.135287	0.134227
